# Speech sound discrimination training improves auditory cortex responses in a rat model of autism

**DOI:** 10.3389/fnsys.2014.00137

**Published:** 2014-08-05

**Authors:** Crystal T. Engineer, Tracy M. Centanni, Kwok W. Im, Michael P. Kilgard

**Affiliations:** Cortical Plasticity Laboratory, School of Behavioral and Brain Sciences, The University of Texas at DallasRichardson, TX, USA

**Keywords:** intervention, plasticity, speech, rehabilitation, auditory cortex, autism, valproate

## Abstract

Children with autism often have language impairments and degraded cortical responses to speech. Extensive behavioral interventions can improve language outcomes and cortical responses. Prenatal exposure to the antiepileptic drug valproic acid (VPA) increases the risk for autism and language impairment. Prenatal exposure to VPA also causes weaker and delayed auditory cortex responses in rats. In this study, we document speech sound discrimination ability in VPA exposed rats and document the effect of extensive speech training on auditory cortex responses. VPA exposed rats were significantly impaired at consonant, but not vowel, discrimination. Extensive speech training resulted in both stronger and faster anterior auditory field (AAF) responses compared to untrained VPA exposed rats, and restored responses to control levels. This neural response improvement generalized to non-trained sounds. The rodent VPA model of autism may be used to improve the understanding of speech processing in autism and contribute to improving language outcomes.

## Introduction

Individuals with autism often have significantly impaired language acquisition and social interactions. While typically developing children exhibit a listening preference for speech over non-speech sounds, children with autism frequently exhibit a listening preference for non-speech sounds (Klin, 1991; Kuhl et al., 2005). Cortical responses to speech sounds in individuals with autism are both weaker and delayed compared to typically developing children (Kuhl et al., 2005; Whitehouse and Bishop, 2008; Roberts et al., 2010), particularly in non-primary auditory fields (Lai et al., 2011; Samson et al., 2011). A recent study found that the cortical response to words in 2 year olds with autism accurately predicts language ability at the age of six. Two year old children with autism that exhibited a left rather than right ERP response to words had better language outcomes 4 years later compared to two year old children with autism that exhibited an atypical ERP response to words (Kuhl et al., 2013). Extensive early intervention therapy in young children with autism has been shown to improve language function, IQ, and adaptive behavior (McEachin et al., 1993; Dawson et al., 2010; Klintwall et al., 2013). This improved outcome following intervention is also associated with faster and stronger cortical responses (Russo et al., 2010; Dawson et al., 2012).

Autism has multiple genetic and environmental causes. For example, prenatal exposure to the antiepileptic drug valproic acid (VPA) causes epigenetic changes (Milutinovic et al., 2007) that increase the risk for both autism and language impairment (Christensen et al., 2013; Meador and Loring, 2013). Both expressive language and language comprehension are impaired in children prenatally exposed to VPA (Meador et al., 2011; Shallcross et al., 2014). The dose of VPA that children were exposed to during the first trimester strongly predicts language scores: children who received a higher dose of VPA had a larger language impairment compared to children who received a lower dose of VPA (Nadebaum et al., 2011). Although no animal model can capture all of the aspects of autism, the well-established VPA model exhibits many behavioral and neural abnormalities observed in autism. For example, rodents prenatally exposed to VPA exhibit reduced social interactions, delayed motor development, improved learning on simple tasks, disturbed emotional responses, increased repetitive behaviors, and reduced ultrasonic vocalizations (Schneider and Przewlocki, 2005; Schneider et al., 2007; Gandal et al., 2010; Edalatmanesh et al., 2013; Roullet et al., 2013). These behavioral abnormalities are associated with many neural abnormalities, including altered synaptic plasticity, connectivity, and excitability (Rinaldi et al., 2008; Silva et al., 2009). Abnormal neural properties have been documented in cortex, hippocampus, amygdala, cerebellum and brainstem (Ingram et al., 2000; Markram et al., 2008; Gandal et al., 2010; Mychasiuk et al., 2012; Martin and Manzoni, 2014). VPA exposed rats exhibit impaired neural processing of speech sounds. Speech responses in the anterior auditory field (AAF) of VPA exposed rats are both slower and weaker than normal (Engineer et al., 2014a). We hypothesize that the degraded cortical representation of speech will interfere with the learning of a simple speech discrimination task and that long term speech training in VPA exposed rats will strengthen cortical responses to speech sounds and restore speech sound discrimination ability to normal.

## Materials and methods

We trained 21 rats to discriminate between consonants and vowels (*n* = 10 VPA exposed speech trained rats; *n* = 11 saline exposed speech trained rats), and recorded AAF responses to speech sounds following training in a subset of these rats (*n* = 5 VPA speech trained rats; *n* = 4 saline speech trained rats). The University of Texas at Dallas Institutional Animal Care and Use Committee approved all protocols and recording procedures.

### Valproic acid exposure

Pregnant rats received an intraperitoneal injection of either saline or 600 mg/kg sodium valproate (Sigma Aldrich) on embryonic day 12.5 (Schneider and Przewlocki, 2005; Banerjee et al., 2013; Favre et al., 2013). Male offspring were used for behavioral training and neurophysiology experiments (Schneider et al., 2008).

### Speech stimuli

The speech sounds used in this study were identical to the sounds used in our previous studies (Engineer et al., 2008, 2014c; Centanni et al., 2013; Perez et al., 2013). All sounds were consonant-vowel-consonant words spoken by a female native English speaker, and the sound set included the words: “bad”, “chad”, “dad”, “dead”, “deed”, “dood”, “dud”, “gad”, “sad”, “shad”, and “tad”. Sounds were shifted into the rat hearing range using the STRAIGHT vocoder (Kawahara, 1997) and intensity adjusted so that the loudest 100 ms of the vowel was presented at 60 dB SPL. Spectrograms, power spectrums, and acoustic cue measurements for these same sounds have been published previously (Engineer et al., 2008, 2013; Perez et al., 2013).

### Speech training

Rats were trained to discriminate speech sounds by consonant (e.g., “dad” vs. “bad”) or vowel (e.g., “dad” vs. “deed”). The speech training procedure used was identical to our previous studies (Engineer et al., 2008, 2014c; Perez et al., 2013). Each rat was initially trained to press a lever to receive a sugar pellet reward (45 mg, Bio-Serv). Rats had to independently press the lever 100 times in a session for two sessions to advance to the next stage of training. Rats were then trained to press the lever in response to hearing the target sound (e.g., “dad”). They initially had to press the lever within 8 s of hearing the target sound, but this window was decreased to 3 s through the course of training. Rats stayed on this stage of training until they reached a criteria of a *d*′ ≥ 1.5 for 10 sessions. Rats were then trained to press the lever for the target sound (e.g., “dad”), and refrain from lever pressing to all non-target sounds (e.g., “bad”, “gad”, “sad”, and “tad” for the consonant task and “dead”, “deed”, “dood”, and “dud” for the vowel task). For the consonant discrimination task, some rats were trained to press the lever for the sound “dad” and refrain from pressing for the sounds “bad”, “gad”, “sad”, and “tad”; other rats were trained to press the lever for the sound “bad” and refrain from pressing for the sounds “dad”, “tad”, “gad”, and “sad”. For the vowel discrimination task, some rats were trained to press the lever for the sound “dad” and refrain from pressing for the sounds “dead”, “dud”, “deed”, and “dood”; other rats were trained to press the lever for the sound “deed” and refrain from pressing for the sounds “dad”, “dead”, “dud”, and “dood”. Some rats trained on the consonant discrimination task first, while other rats trained on the vowel task first. Each discrimination task lasted for a period of 3 weeks. Pressing the lever in response to a target sound resulted in a sugar pellet reward, while pressing the lever in response to a non-target sound resulted in the program pausing and the training booth lights extinguishing for a period of 6 s.

### Physiology

Within 24 h after the last day of speech sound discrimination training, auditory cortex recordings were obtained from speech trained rats. The recording procedure used was identical to our previous studies (Engineer et al., 2008; Centanni et al., 2013; Perez et al., 2013). Auditory cortex responses from 92 AAF recording sites were recorded in 5 (of the 10) VPA exposed speech trained rats (VPA speech trained group) were compared to 106 AAF recording sites recorded in 11 VPA exposed untrained rats (VPA untrained group), 116 AAF recording sites recorded in six saline exposed untrained rats (Saline untrained group), and 88 AAF recording sites recorded in 4 (of the 11) saline exposed speech trained rats (Saline speech trained group). Neural recordings from the remaining VPA exposed and saline exposed speech trained rats were made in other auditory fields (primary auditory cortex, ventral auditory field, and posterior auditory field), and are not included in this study. Rats were anesthetized with sodium pentobarbital (50 mg/kg), and were provided with supplemental doses of pentobarbital throughout the experiment (8 mg/mL). A tracheotomy was performed and humidified air was provided to facilitate breathing throughout the experiment. A cisternal drain was performed to prevent swelling, and a craniotomy and durotomy were performed over right auditory cortex. Body temperature was maintained with a heating pad at 37°C, and a lactated Ringer’s and dextrose solution was provided throughout the experiment to maintain hydration. Multiunit responses were recorded from right AAF using four Parylene-coated tungsten microelectrodes (FHC, 1–2 MΩ) lowered simultaneously. Recordings were made immediately following the surgery, and recording sessions lasted an average of 10.8 ± 0.9 h (range 3–18.5 h). Recording sites were chosen to evenly sample AAF while avoiding blood vessels. Tucker-Davis speakers (FF1), hardware (RA16 and RX5), and software (SigGen and Brainware) were used for sound generation and data acquisition. Tone frequency intensity tuning curves (25 ms tones ranging from 1–32 kHz in 0.125 octave steps and ranging from 0–75 dB SPL in 5 dB steps) were obtained to determine the characteristic frequency of each auditory cortex site. Trains of six noise bursts (1–32 kHz range, 10 Hz) and each of the speech sounds used during speech discrimination training were also presented at each recording site (20 repeats each).

### Data analysis

Consonant and vowel discrimination performance was quantified in units of percent correct, which is defined as the average of the correct lever presses to target sounds and the correct rejections of non-target sounds.

AAF recording sites were defined based on relative location, a distinctive tonotopy of low to high characteristic frequencies from anterior to posterior, and fast response latencies to tones (Polley et al., 2007; Centanni et al., 2013). The response strength to speech sounds was quantified as the number of spikes evoked in the first 40 ms of consonant onset. The onset latency to speech sounds was quantified as the time point where the neural response was three standard deviations above the spontaneous firing rate, and the peak latency to speech sounds was quantified as the time point of maximum neural firing.

Neural speech sound classification accuracy was measured using a PSTH-based nearest neighbor classifier to quantify neural discrimination using spike timing (40 1-ms bins) from single sweeps of neural activity (Foffani and Moxon, 2004; Schnupp et al., 2006; Engineer et al., 2008; Centanni et al., 2013; Perez et al., 2013). The response of each trial was compared with the average activity pattern evoked by each of two speech sounds. Euclidean distance was used to determine the similarity between each single response trial and the average activity pattern generated by each of the two speech sounds. The classifier assigned the single response trial to the speech sound whose average activity pattern it most closely resembled. Vector strength was used to quantify the degree of synchronization between the noise bursts and the neural response (Lu et al., 2001; Bao et al., 2004; Centanni et al., 2014a). A vector strength value of 0 indicates no synchronization, while a value of 1 indicates perfect synchronization. Vector strength is calculated as:

(1) $$\text{VS}=\frac{1}{n}\sqrt{x^{2}+y^{2};}\;x=\sum_{i=1}^{n}\cos\theta_{i};\;y=\sum_{i=1}^{n}\sin\theta_{i}\theta_{i}=2\pi \frac{t_{i}}{T}$$

where *t_i_* is the timing of the *i*th action potential, *T* is the interstimulus interval between noise bursts, and *n* is the total number of action potentials. The tone threshold for each recording site was defined as the lowest intensity that evoked a response at the characteristic frequency. The bandwidth for each recording site was defined as the frequency range (in octaves) that evoked a response 40 dB above the tone threshold.

## Results

### Speech sound discrimination

**Figure 1:**
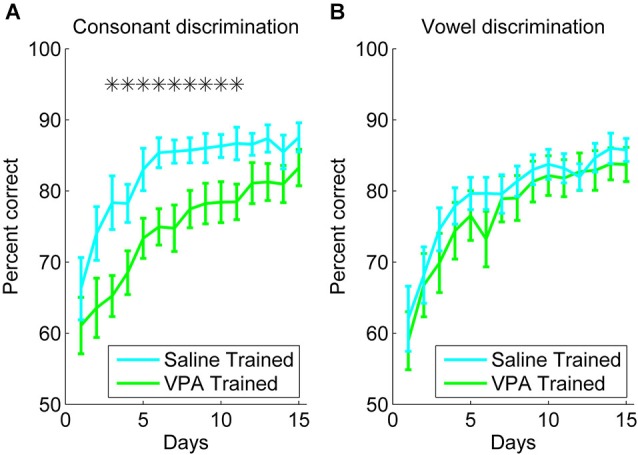
**VPA exposed rats are impaired at consonant discrimination, but not vowel discrimination. (A)** Consonant discrimination time courses for VPA exposed (*n* = 10 rats; green line) and saline exposed rats (*n* = 10 rats; cyan line). Error bars indicate s.e.m. across rats. Stars indicate time points where VPA exposed rats perform significantly worse than saline exposed rats (*p* < 0.05). **(B)** Vowel discrimination time courses for VPA exposed (*n* = 10 rats) and saline exposed rats (*n* = 11 rats).

Rats were trained to discriminate between speech sounds differing by consonant (e.g., “dad” vs. “bad”) or vowel (e.g., “dad” vs. “deed”). VPA exposed rats were significantly impaired at consonant discrimination, but not vowel discrimination. For the consonant discrimination task, rats were trained to press the lever in response to the target speech sound, and refrain from lever pressing in response to the non-target speech sounds. Over 3 weeks of training, both the VPA exposed and saline exposed rats responded more accurately over time (*F*_(14, 210)_ = 30.73, *p* < 0.0001, two-way repeated measures ANOVA, Figure 1A). However, the VPA exposed rats were significantly more impaired at consonant discrimination compared to saline exposed rats (*F*_(1, 210)_ = 5.72, *p* = 0.03, two-way repeated measures ANOVA, Figure 1A). There was not a significant interaction between rat group and time for the consonant discrimination task (*F*_(14, 210)_ = 0.72, *p* = 0.76, two-way repeated measures ANOVA, Figure 1A).

For the vowel discrimination task, rats were trained to press the lever in response to the target speech sound (for example, “dad”), and refrain from lever pressing in response to the non-target speech sounds (for example, “dead”, “dud”, “deed”, and “dood”). Over 3 weeks of training, both the VPA exposed and saline exposed rats responded more accurately over time (*F*_(14, 210)_ = 26.39, *p* < 0.0001, two-way repeated measures ANOVA, Figure 1B). In contrast to the consonant discrimination task, the VPA exposed rats were unimpaired at vowel discrimination compared to saline exposed rats (*F*_(1, 210)_ = 0.03, *p* = 0.86, two-way repeated measures ANOVA, Figure 1B). There was not a significant interaction between rat group and time for the vowel discrimination task (*F*_(14, 210)_ = 0.38, *p* = 0.98, two-way repeated measures ANOVA, Figure 1B). Our finding that VPA exposed rats are impaired at consonant, but not vowel, discrimination is consistent with behavioral findings in individuals with autism, showing intact processing of spectral information, but impaired processing of rapid temporally complex information (Samson et al., 2011). We predicted that AAF responses to speech sounds would be enhanced following extensive speech sound training.

### Neural responses to speech sounds after training

**Figure 2:**
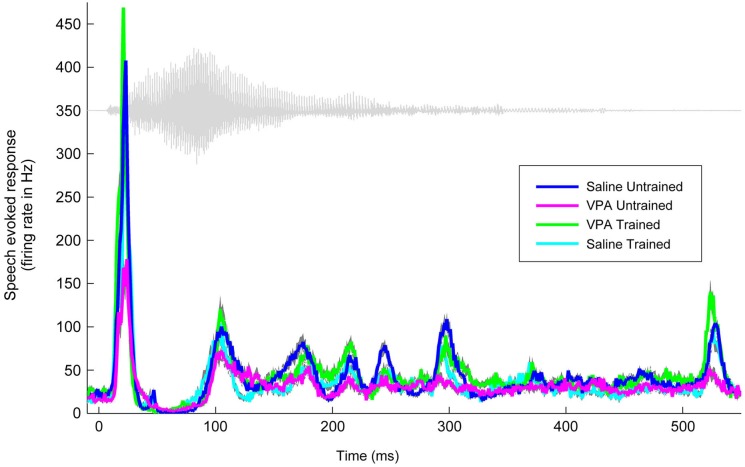
**Speech training enhances the neural response to the speech sound “gad”**. The weakened response to “gad” in AAF in VPA untrained rats (*n* = 11 rats; magenta line) is normalized in VPA speech trained rats (*n* = 5 rats; green line), and is compared to saline untrained rats (*n* = 6 rats; blue line) and saline speech trained rats (*n* = 4 rats; cyan line). Gray shading indicates s.e.m. across recording sites. The waveform for the speech sound “gad” is plotted in gray.

**Figure 3:**
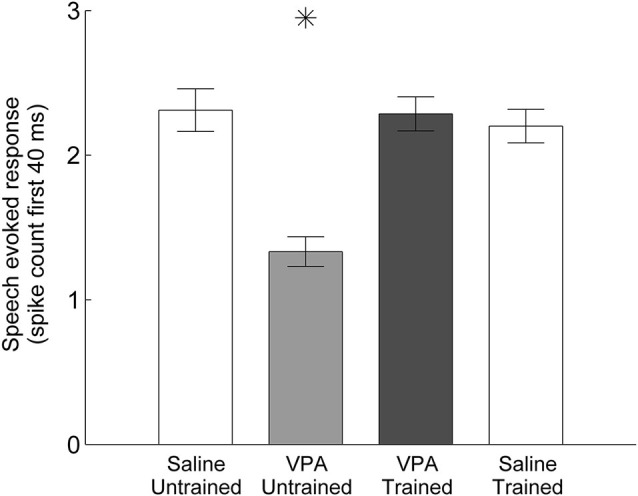
**Mean spike count in response to speech sounds is restored in VPA speech trained rats compared to VPA untrained rats**. The number of driven spikes is the average of the 40 ms onset response across the 11 speech sounds presented. Error bars indicate s.e.m. across AAF recording sites. Asterisks indicate groups that are statistically significant compared to VPA speech trained rats (*p* < 0.05).

We have previously documented the impaired AAF responses to speech sounds in rats that were prenatally exposed to VPA (Engineer et al., 2014a). Speech training enhanced the AAF response to speech sounds in VPA exposed speech trained rats (Figure 2). Following speech training, the response strength to speech sounds significantly improved in AAF in VPA speech trained rats (1.3 ± 0.1 spikes VPA untrained vs. 2.3 ± 0.1 spikes VPA speech trained, *p* < 0.0001, Figure 3). The response strength in speech trained VPA rats was indistinguishable from saline untrained rats (2.3 ± 0.1 spikes VPA speech trained vs. 2.3 ± 0.1 spikes saline untrained, *p* = 0.89, Figure 3).

**Figure 4:**
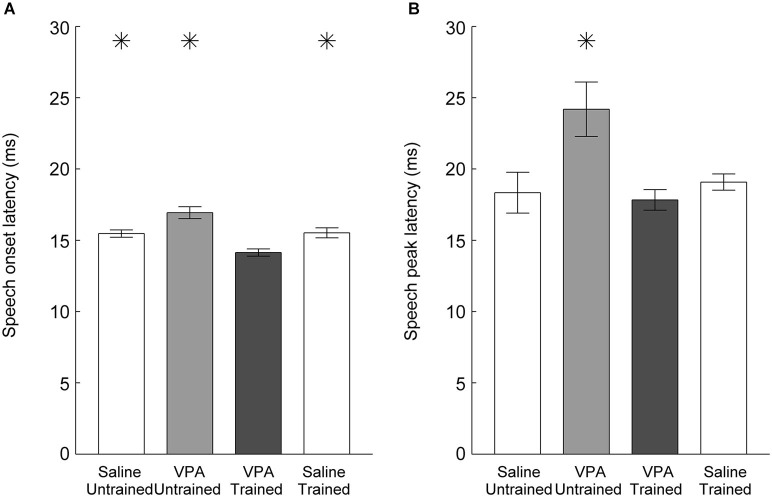
**Speech sound response latency in AAF is normalized following speech training. (A)** The slower onset latency to speech sounds observed in VPA untrained rats is significantly faster following speech training. Error bars indicate s.e.m. across recording sites. **(B)** The slower peak latency to speech sounds observed in VPA untrained rats is restored following speech training. Asterisks indicate groups that are statistically significant compared to VPA speech trained rats (*p* < 0.05).

In addition to weaker responses, untrained VPA exposed AAF responses to speech sounds are delayed (Engineer et al., 2014a). Following speech training, the onset latency to speech sounds was significantly faster in VPA speech trained rats compared to VPA untrained rats (16.9 ± 0.4 ms VPA untrained vs. 14.1 ± 0.3 ms VPA speech trained, *p* < 0.0001, Figure 4A). The peak latency was also significantly improved in VPA speech trained rats compared to VPA untrained rats (24.2 ± 1.9 ms VPA untrained vs. 17.8 ± 0.7 ms VPA speech trained, *p* = 0.003, Figure 4B). Extensive speech training improved both the response strength and response latency deficits observed in VPA exposed rats.

**Figure 5:**
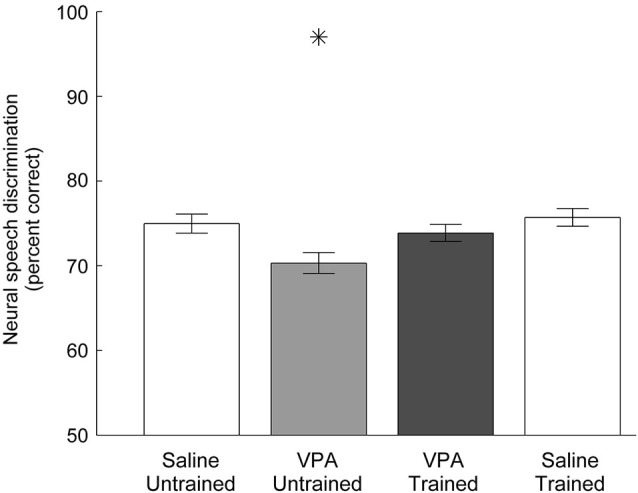
**Neural discrimination of speech sounds in AAF is restored in VPA speech trained rats**. The classifier percent correct impairment in VPA untrained rats is restored in VPA speech trained rats. The classifier uses the 40 ms onset response with 1 ms precision to discriminate between pairs of consonants. Error bars indicate s.e.m. across AAF recording sites. Asterisks indicate groups that are statistically significant compared to VPA speech trained rats (*p* < 0.05).

As expected from the weaker and slower AAF responses to speech, VPA exposed neurons are less able to discriminate between pairs of speech sounds (Engineer et al., 2014a). Using a nearest-neighbor classifier, VPA exposed AAF neurons were significantly less able to correctly identify the consonant that produced each neural activity pattern. Neural discrimination of speech sounds was restored in speech trained VPA exposed rats. The classifier percent correct impairment in VPA untrained rats (70 ± 1 percent correct, *p* = 0.005 compared to saline untrained rats) was restored to saline untrained levels in VPA speech trained rats (75 ± 1 percent correct saline untrained vs. 74 ± 1 percent correct VPA speech trained, *p* = 0.48, Figure 5). Neural discrimination was significantly improved in VPA exposed rats following speech training (VPA untrained vs. VPA speech trained, *p* = 0.03, Figure 5). These findings suggest that improving both the response strength and the temporal precision of the response is sufficient to restore the unique spatiotemporal activity patterns produced by each speech sound in saline untrained rats.

### Neural responses to non-speech sounds after training

**Figure 6:**
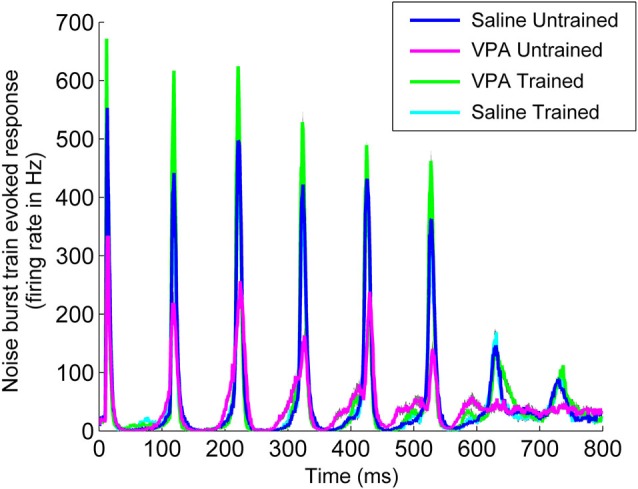
**Speech training enhances the neural response to a 10 Hz train of six noise bursts**. The weak response to noise bursts in AAF in VPA untrained rats (magenta line) is enhanced in VPA speech trained rats (green line), and is compared to saline untrained rats (blue line) and saline speech trained rats (cyan line). Gray shading indicates s.e.m. across recording sites.

**Figure 7:**
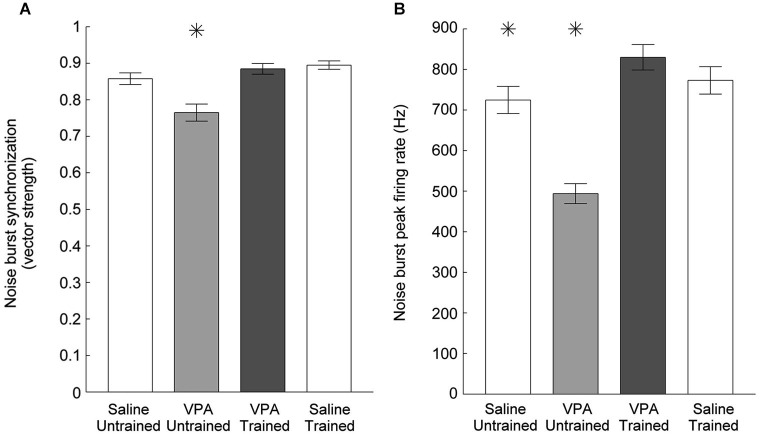
**Cortical phase locking is enhanced in VPA speech trained rats. (A)** The decreased degree of synchronization in VPA untrained rats compared to saline untrained rats is restored following speech training. Error bars indicate s.e.m. across recording sites. Asterisks indicate groups that are statistically significant compared to VPA speech trained rats (*p* < 0.05). **(B)** The mean peak firing rate across all six noise bursts is enhanced following speech training.

Impaired temporal processing has been previously documented in VPA exposed rats (Gandal et al., 2010; Engineer et al., 2014a). VPA exposed rats had both decreased synchronization and decreased response strength to rapidly presented stimuli. In addition to presenting speech sounds during the neural recordings following speech training, we also presented a 10 Hz train of noise burst stimuli and recorded AAF responses (Figure 6). Following speech training, synchronization was significantly enhanced in VPA speech trained rats compared to VPA untrained rats (0.77 ± 0.02 VPA untrained vs. 0.89 ± 0.2 VPA speech trained, *p* < 0.0001, Figure 7A). The average peak firing rate was also significantly improved in VPA speech trained rats compared to VPA untrained rats (493.9 ± 24.5 Hz VPA untrained vs. 829.8 ± 31.4 ms VPA speech trained, *p* < 0.0001, Figure 7B). Extensive speech training improved the temporal processing deficits observed in VPA exposed rats.

**Figure 8:**
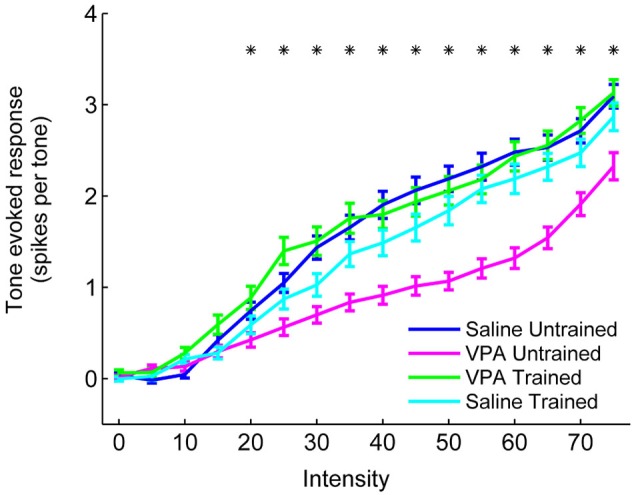
**The response strength to tones is enhanced in VPA speech trained rats**. VPA untrained rats evoke fewer spikes per tone in AAF compared to saline untrained rats, and this deficit is reversed in VPA speech trained rats. The number of spikes evoked per tone is presented at a range of intensities (0–75 dB). Responses to tones within 1 octave of each site’s characteristic frequency were averaged together. Error bars indicate s.e.m. across recording sites. Asterisks indicate intensities that are statistically significant between VPA untrained rats and VPA speech trained rats (*p* < 0.0031, Bonferroni correction).

In addition to impaired temporal processing, we have also observed a reduced response to tones in VPA exposed rats (Engineer et al., 2014a). AAF responses to tones were reduced in VPA exposed rats at intensities between 20 and 70 dB (Figure 8). Following speech training, responses to tones were restored to saline untrained response strengths across the intensity range (*p* < 0.0031, Bonferroni correction, Figure 8). Additionally, VPA exposed rats needed a higher intensity tone in order to evoke a neural response (26.6 ± 1.4 dB threshold in VPA untrained rats vs. 21.7 ± 0.9 dB threshold in saline untrained rats, *p* = 0.003). This intensity threshold was restored to saline untrained levels following speech training (22.3 ± 1.5 dB threshold in VPA speech trained rats, *p* = 0.72 compared to saline untrained rats; *p* = 0.03 compared to VPA untrained rats). While VPA exposed rats also exhibited an increase in the range of frequencies that evoked a response 40 dB above threshold (3.1 ± 0.1 octave bandwidth in VPA untrained rats vs. 2.8 ± 0.1 octave bandwidth in saline untrained rats, *p* = 0.04), this frequency range was not restored following speech training (3.2 ± 0.1 octave bandwidth in VPA speech trained rats, *p* = 0.002 compared to saline untrained rats; *p* = 0.61 compared to VPA untrained rats). In addition to improving speech sound discrimination and the AAF representation of speech sounds, extensive speech training generalized to non-trained sounds and strengthened the AAF response to tones and temporally modulated sounds.

### Training does not enhance responses in unexposed rats

Our results demonstrate that training improves AAF responses in VPA speech trained rats. However, it is possible that training also improves responses in rats not exposed to VPA, such that VPA speech trained rats remain impaired relative to saline speech trained rats. To test this hypothesis, we quantified AAF responses to speech and non-speech sounds in a subset of saline exposed speech trained rats (*n* = 4 out of the 11 saline speech trained rats). Our results indicate that speech training did not enhance the response strength or response latency to speech and non-speech sounds in saline speech trained rats. Saline speech trained rats responded to speech sounds with 2.2 ± 0.1 spikes, which was not significantly different from saline untrained (2.3 ± 0.1 spikes, *p* = 0.57) or VPA speech trained rats (2.3 ± 0.1 spikes, *p* = 0.61, Figure 3). Saline speech trained rats responded to speech sounds with an onset latency of 15.5 ± 0.4 ms, which was not significantly different from saline untrained rats (15.5 ± 0.3 ms, *p* = 0.91) and was significantly slower than VPA speech trained rats (14.1 ± 0.3 ms, *p* = 0.002, Figure 4A). Saline speech trained rats responded to speech sounds with a peak latency of 19.1 ± 0.6 ms, which was not significantly different from saline untrained rats (18.3 ± 1.4 ms, *p* = 0.67) or VPA speech trained rats (17.8 ± 0.7 ms, *p* = 0.18, Figure 4B). As expected since AAF responses to speech sounds were not faster and stronger, neural discrimination of speech sounds in saline speech trained rats (76 ± 1 percent correct) was not significantly different from saline untrained (75 ± 1 percent correct, *p* = 0.64) or VPA speech trained rats (74 ± 1 percent correct, *p* = 0.2, Figure 5).

Speech training also did not enhance responses to non-speech sounds in saline speech trained rats. Synchronization to noise bursts in saline speech trained rats (0.89 ± 0.01) was not significantly different from saline untrained rats (0.86 ± 0.02, *p* = 0.08) or VPA speech trained rats (0.89 ± 0.02, *p* = 0.58, Figure 7A). The response strength to noise burst sounds in saline speech trained rats (772.9 ± 33.8 Hz) was not significantly different from saline untrained rats (724.6 ± 33.4 Hz, *p* = 0.32) or VPA speech trained rats (829.8 ± 31.4 Hz, *p* = 0.22, Figure 7B). Responses to tones in saline speech trained rats were not significantly different across the intensity range compared to both saline untrained and VPA speech trained rats (*p* < 0.0031, Bonferroni correction, Figure 8). These results provide further support that training ameliorates the degraded AAF responses to speech and non-speech sounds caused by *in utero* exposure to VPA.

## Discussion

Delayed language acquisition and social impairments are often observed in children with autism. We have previously reported impaired AAF responses to speech sounds in the prenatal VPA exposure rodent model of autism. In this study, we extend that finding by showing that prenatal VPA exposure also significantly impairs speech sound discrimination ability. VPA exposed rats were less able to perform a consonant discrimination task, while vowel discrimination was unimpaired. Extensive speech sound training improved both consonant discrimination performance and AAF responses to speech sounds. Responses were faster and stronger following speech training, and this neural improvement also generalized to non-trained sounds. The weak and slow cortical responses to speech sounds and impaired speech discrimination may limit language acquisition in children with autism. Impaired communication could exacerbate many of the other symptoms of autism; improved communication likely plays a major role in their alleviation (Dawson et al., 2010). Although we did not test other symptoms of autism, we expect that speech training would not generalize to improve repetitive behavior, social, and other impairments, which would support proposals that behavioral therapy should address each of the behavioral impairments. Intensive early intervention that makes cortical responses faster and stronger and improves speech discrimination performance may also support improved language acquisition and social interactions in children with autism.

### Speech sound discrimination

In this study, we documented that VPA exposed rats have impaired consonant discrimination, but normal vowel discrimination. This finding is consistent with both the auditory and visual system literature in individuals with autism showing normal or enhanced performance on tasks using simple stimuli, but impaired performance on tasks using complex stimuli (Bertone et al., 2005; Marco et al., 2011; Samson et al., 2011). For example, children with autism often have enhanced pitch discrimination, but are less able to discriminate between speech sounds in background noise (O’Connor, 2012). While studies documenting neural responses to simple stimuli in individuals with autism have conflicting results, neural responses to spectrotemporally complex stimuli in individuals with autism are consistently impaired (Čeponienė et al., 2003; Marco et al., 2011; O’Connor, 2012).

Studies in both humans and rats have documented that consonants and vowels are processed differently. Our previous study showed that precise action potential timing information is necessary for accurate neural discrimination of consonants, while the neural discrimination of vowels is accomplished with only the average spike rate information (Perez et al., 2013). The finding that VPA exposed rats are impaired at consonant, but not vowel, discrimination matches previous studies showing a rapid temporal processing deficit in individuals with autism (Oram Cardy et al., 2005; Kwakye et al., 2011).

### Non-primary auditory cortex

Many studies have reported intact auditory processing in primary auditory cortex in individuals with autism, but impaired auditory processing in non-primary auditory cortex (Čeponienė et al., 2003; Lai et al., 2011; Samson et al., 2011; Abrams et al., 2013). We have also observed intact auditory processing in primary auditory cortex, but impaired auditory processing in non-primary auditory cortex in the rodent VPA model of autism (Engineer et al., 2014a). The neural responses in this study were recorded from AAF in VPA exposed and saline exposed rats, which is a field known to be important for pattern and temporal discrimination tasks. Deactivating AAF in cats impairs pattern discrimination ability (Lomber and Malhotra, 2008), while removing AAF in rats impairs speech sound discrimination ability (Kudoh et al., 2006). Together, these findings suggest that low-level sensory processing of less complex sounds is preserved in autism, while higher level processing of more complex sounds is impaired.

### Temporal processing deficits

Auditory temporal processing deficits are not unique to autism, and have been observed in other patient populations, such as dyslexia or specific language impairment (Tallal et al., 1997). We have previously demonstrated reduced neural discrimination of speech sounds and increased trial-by-trial variability in the response to speech sounds in primary auditory cortex of a rat model of dyslexia (Centanni et al., 2014a). These rats were impaired at both consonant and vowel discrimination. Extensive speech sound training normalized both discrimination ability and auditory cortex responses to speech (Centanni et al., 2014b). Temporal processing abnormalities have also been observed in both individuals with fragile X syndrome and a rodent model of fragile X syndrome (Frankland et al., 2004). We observed significantly impaired responses to speech, noise bursts, and tones in AAF, primary auditory cortex, and ventral auditory field in the *Fmr1* KO rat model of fragile X syndrome (Engineer et al., 2014b). *Fmr1* KO rats were unimpaired at consonant and vowel discrimination, and weeks of speech training did not improve auditory cortex responses. While the autism, dyslexia, and fragile X syndrome rodent models all exhibit a reduced response strength to noise burst trains, the behavioral and neural ability to discriminate speech sounds varies across the models. More research is needed to help disambiguate the training profiles of different populations.

### Intervention therapy

Our results demonstrate that thousands of speech discrimination trials over many weeks can improve speech discrimination ability and AAF responses to sounds in VPA exposed rats. Thousands of children have been prenatally exposed to the antiepileptic drug valproate, and these children have at least a four-fold increased risk of developing autism (Moore et al., 2000; Rasalam et al., 2005; Christensen et al., 2013; Meador and Loring, 2013). It is estimated that more than 60% of children exposed to VPA require speech therapy or educational support (Moore et al., 2000; Adab et al., 2001; Viinikainen et al., 2006). Clinical trials could be used to determine how well children who have been prenatally exposed to VPA respond to intensive speech therapy and whether such training alters cortical evoked responses.

Our result is consistent with previous studies in children with autism which demonstrate that extensive intervention therapy occurring for 20 or more hours per week over many months drastically improves outcomes. Children who underwent this extensive therapy had an average gain of 18 IQ points and exhibit normalized cortical activity (McEachin et al., 1993; Dawson et al., 2010, 2012). Studies have shown that the mental age at the start of therapy and the extent of the therapy are both important predictors of outcome (Klintwall et al., 2013). In addition, brain responses at age two in children with autism can predict language outcomes at age six after extensive training (Kuhl et al., 2013). It is not yet clear whether training was associated with changes in the spatial distribution of cortical responses to speech sounds in VPA exposed rats. Many studies have documented improved outcomes in adolescents and adults with autism (Piven et al., 1996; Seltzer et al., 2004). Additional studies are needed to determine if there are persistent impairments in auditory processing in untrained VPA exposed rats throughout their lifetime, or if the impairments improve over time without training. Rodent models of autism may prove valuable for understanding why some subtypes of autism benefit from training more than others. For example, while the rodent VPA model represents an environmental cause of autism, additional studies are needed to determine whether speech sound processing is similarly affected in genetic models of autism.

## Conflict of interest statement

The authors declare that the research was conducted in the absence of any commercial or financial relationships that could be construed as a potential conflict of interest.
